# Supplementary material for: Mechanisms governing the pioneering and redistribution capabilities of the non-classical pioneer PU.1
Source: Nat Commun. 2020 Jan 21;11:402. doi: 10.1038/s41467-019-13960-2 (PMC6972792; doi:10.1038/s41467-019-13960-2)
Supplement: Supplementary file 7 — Source data [file 41467_2019_13960_MOESM7_ESM.zip › Source_Data/FigureS2/FigureS2B_MotifScanOutput/KmeansCluster7/homerResults/motif37.similar.html]

motif37

## Information for motif37

A
G
C
T
A
T
C
G
G
T
C
A
A
T
G
C
C
G
A
T
G
A
T
C
C
T
G
A
A
G
C
T
A
G
C
T
C
G
T
A
  
Reverse Opposite:  

G
C
A
T
T
C
G
A
T
C
G
A
A
G
C
T
C
T
A
G
C
G
T
A
T
A
C
G
A
C
G
T
T
A
G
C
C
T
G
A
  

|  |  |
| --- | --- |
| p-value: | 1e-35 |
| log p-value: | -8.120e+01 |
| Information Content per bp: | 1.718 |
| Number of Target Sequences with motif | 247.0 |
| Percentage of Target Sequences with motif | 7.69% |
| Number of Background Sequences with motif | 1307.7 |
| Percentage of Background Sequences with motif | 2.99% |
| Average Position of motif in Targets | 109.4 +/- 54.8bp |
| Average Position of motif in Background | 98.7 +/- 67.3bp |
| Strand Bias (log2 ratio + to - strand density) | 0.1 |
| Multiplicity (# of sites on avg that occur together) | 1.07 |
| Motif File: | file (matrix) reverse opposite |

### Similar de novo motifs found

|  |  |  |  |  |  |  |  |
| --- | --- | --- | --- | --- | --- | --- | --- |
| Rank | Match Score | Redundant Motif | P-value | log P-value | % of Targets | % of Background | Motif file |
| 1 | 0.850 | A G C T A T C G C T G A T A C G C G A T G T A C C T G A A C G T T G C A | 1e-25 | -58.318115 | 8.16% | 3.88% | motif file (matrix) |
| 2 | 0.780 | A G C T A C T G C G T A A C T G A C G T A G T C C T G A | 1e-23 | -54.017342 | 16.36% | 10.29% | motif file (matrix) |
| 3 | 0.858 | A C G T A C T G G T C A T A C G A C G T A G T C C T G A A G C T | 1e-18 | -43.261294 | 7.17% | 3.66% | motif file (matrix) |
